# Supplementary material for: Are carbon emissions trading and green financial instruments synergistic? -Comprehensive quantitative research based on content analysis
Source: PLoS One. 2024 Mar 7;19(3):e0298601. doi: 10.1371/journal.pone.0298601 (PMC10919601; doi:10.1371/journal.pone.0298601)
Supplement: S1 Data — (ZIP) [file pone.0298601.s001.zip › Data/地级市Z_F工作报告.zip!/╣π╢1⁄2/╔╟╬▓╩╨/└┤╫╘╣1⁄2╓┌║┼í╛┬φ┐╦╩2╛▌═°í┐_2017.docx]

各位代表：

我代表市人民政府向大会作政府工作报告，请予审议，并请政协各位委员和其他列席人员提出意见。

过去五年工作回顾

市六届人大一次会议以来的五年，是汕尾坚定向西融珠发展、发展动力持续增强的五年，是积极应对各种挑战、各项事业不断发展的五年。五年来，在省委、省政府和市委的坚强领导下，全市上下全面贯彻党的十八大和十八届三中、四中、五中、六中全会精神，深入贯彻习近平总书记系列重要讲话精神，主动把握和引领经济发展新常态，抢抓机遇、真抓实干，圆满完成了市六届人大一次会议确定的各项目标任务。

——过去五年，是经济保持中高速增长、迈向中高端水平的五年。我们坚持创新引领，推动产业转型升级，经济持续健康发展，质量效益不断提高。2016年预计全市地区生产总值810亿元，增长7.0%，五年年均增长9.9%;人均生产总值26740元，年均增长9.3%；固定资产投资673.6亿元，年均增长15.4%；社会消费品零售总额535亿元，年均增长10.8%；一般公共预算收入30.78亿元，比2015年增长13.8%，非税收入占比35.4%,一般公共预算支出206.65亿元；城镇和农村常住居民人均可支配收入分别为22327元、12261元，年均增长9.2%和9.6%；金融机构本外币存款余额744.28亿元，年均增长14.8%；三次产业结构为15.5:44.9:39.6；实体经济加快发展，2016年新培育“四上”企业115家，规模以上工业增加值年均增长16.1%；国家高新技术企业12家，比2015年增长200%；高技术制造业增加值占规模以上工业增加值比重提高到21.2%，R&D经费支出年均增长17.4%，工业技改投资年均增长7.7%，专利申请量和授权量分别年均增长26.6%和28.1%；第三产业增加值年均增长9.2%；现代农业和海洋渔业加快发展，引进海亮、宝莲生物、中汕实业等10个农业龙头项目。

——过去五年，是向西融珠打开新局面、产业共建取得新成效的五年。我们认真贯彻春华书记“心无旁骛向西发展，主动全面融入珠三角”新要求，深入实施向西融珠战略，全面参与深莞惠+汕尾河源“3+2”经济圈建设，积极探索产业共建新模式，投入园区建设资金69.5亿元，2015、2016年共征地4.19万亩，其中获省批准2万亩，供地1.5万亩。制定入园产业投入、产出、用地等刚性约束标准，出台一系列产业扶持政策和降低企业落户制度性交易成本的措施。深汕特别合作区上升为省级发展战略，红草产业园被认定为省级高新技术开发区，海丰、陆丰、陆河3个产业园纳入省级产业园区管理，红海湾成为国家级海洋公园和省滨海旅游产业示范园区，“1+4+1”产业园区新格局基本形成。坚持“内塑形象、外引资源”，扩大招商队伍，创新招商机制，共引进项目395个，其中投产109个，在建161个，比亚迪新能源汽车、信利TFT5代线、华为云服务、国信通科技、海王制药、上海莱士等一批产值超十亿、超百亿元项目纷纷落户。

——过去五年，是扩容提质加快推进、城镇建设成效显著的五年。我们全力推进城市扩容提质，城镇化率提升至55.08%。加快新区三大起步区建设，金町湾滨海旅游度假区保利一、二期项目进展顺利，中央商务区站前广场建成运营，周边配套道路加快建设，保利大都会、碧桂园时代城、农商行大厦及综合交通枢纽中心等成功落户。推进交通基础设施建设，累计投入资金138.8亿元，是前五年的2.2倍。新改建公路1528公里，全市行政村100%实现公路硬底化。厦深铁路、潮惠高速建成通车，广汕铁路、兴汕高速建设全面启动。红海西路成功破解拆迁难题即将全线贯通，汕尾大道综合改造及两侧环境整治临近竣工，完成工业大道东段、新湖大道南段、金湖路西段、腾飞路等建设，打通文德路、成业路、永和路等7条断头路，站前路、金湖路东段、东城路、金鹏路开工建设，工业大道西即将动工，珠东快速前期工作加快推进，完成海丰县红城大道、二环路，陆丰市东海大道，省道335线陆河河口至新田段等改建工程。大力整治市容环境，大规模持续集中清理城乡垃圾，城市卫生环境得到根本改观。严厉查处违法建设，市区拆除各类违法建设、违章搭建5000多处，清理占道经营6000多处，实现违法建设“零增量”。完成7.2公里环品清湖路亮化美化工程，公共停车场、公厕、群众健身路径等便民设施逐步完善。实施村村通自来水工程，农村自来水普及率达89.2%。建成省级宜居示范镇、村20个，绿化美化乡村示范点610个，海丰县生态科技城、大湖镇和陆河县水唇镇列入省新型城镇化“2511”试点。

——过去五年，是生态环境得到保护、绿色发展持续推进的五年。我们大力推进生态文明建设，完成主要污染物减排任务，超额完成“黄标车”和老旧车淘汰任务，全面启动0－50公里纯电动绿色公交全覆盖，投放纯电动公交车450辆，空气质量持续位居全省前列。实行最严格水资源管理制度，中小河流得到有效治理，公平、龙潭水库和螺河水资源保护成效明显，城市集中式饮用水源水质全部达标，水资源保护工作在全省“十二五”期末考核中获优秀等次。全面开展新一轮绿化广东大行动，人工造林114万亩，建设生态景观林带413公里、森林公园23个、湿地公园5个，森林覆盖率达55.27%，成功创建“广东省园林城市”，陆河县被纳入国家重点生态功能区。建成污水处理厂7座，市生活垃圾无害化处理中心首期工程投入运营，初步建立“户收集、村集中、镇转运、县处理”的农村生活垃圾收运处理体系，城镇生活污水集中处理率、生活垃圾无害化处理率分别达87.5%和88.5%。海湾整治和生态岛礁建设加快推进，被列为全国首批蓝色海湾整治行动城市。

——过去五年，是改革开放不断深化、发展活力明显增强的五年。我们坚持以改革破难题，以开放促发展,不断深化行政审批制度改革，累计取消和调整354项行政审批事项，编制政府部门权责清单，全面清理非行政许可审批事项，大力推广“一门式、一网式”政府服务模式改革，2590项政务服务事项实现网上办理。完成“营改增”试点改革。扎实推进供给侧结构性改革，国有关停类“僵尸企业”实现市场出清20户，房地产去库存37.5万平方米，市汽运公司等完成改制。加快投融资改革，新组建交通投资、水务投资2个投融资平台，千方百计争取到一批国家政策性优惠贷款和政府债券资金，一批项目列入省PPP推介项目库，海丰县成功发行企业债6亿元，市12亿元企业债发行工作进展顺利。商事制度改革稳步推进，已登记各类市场主体12.3万个。深入实施农村综合改革，建成57个镇级农村集体资产资源交易平台，完成4230亩农村土地承包经营权确权登记颁证工作，农村普惠金融“村村通”工作取得阶段性成果。对外开放继续深化，外贸进出口总值累计完成174亿美元，年均增长11%，汕尾港口岸红海湾港区正式对外开放，海丰小漠港区、陆丰甲湖湾港区列入国家口岸发展规划，华润海丰电厂码头口岸试运行。

——过去五年，是社会事业全面发展、人民生活水平不断提高的五年。我们着力建机制、补短板、兜底线，办好各项民生实事，民生领域累计投入541.3亿元，占公共财政支出比重的73.4%。新增城镇就业23.2万人，转移农村劳动力21.2万人，城镇登记失业率控制在3.0%以内，五大险种参保总人次达562.14万。以教育“创强争先建高地”为总抓手，教育普及程度有新提高，学前三年教育毛入园率、高中阶段教育毛入学率分别比五年前提高30个百分点和17个百分点，小学、初中学龄人口入学率达100%，初中辍学率控制在2.5%以下。校长、教师队伍建设进一步加强，华南师大附中汕尾学校、华中师大海丰附属学校等民办教育健康发展，成功创建成为义务教育基本均衡市和教育强市。市直公立医院改革有序推进，深化与中信集团合作，创新市直公立医院管理与运营机制。逸挥医院成为首家三甲医院。每千人床位数、执业（助理）医师数、注册护士数比2011年分别增长18%、20%、50%，全市城乡居民基本医疗保险参保率保持在98%以上，大病保险覆盖率达100％，整体医疗服务保障能力得到提升。汕尾被纳入国家贫困革命老区扶持范围，我们加强对接工作，义务教育补贴、高铁和高速公路资本金、蓝色海湾整治等一批扶持政策落地，2016年获得国家、省扶持资金11亿元，老区工作获省表彰。完成保障性住房8359套（户）、棚户区改造1587户。全面完成两轮“规划到户、责任到人”扶贫开发任务，帮扶413个贫困村、33501户贫困户，完成农村危房改造40824户和“两不具备”村庄1729户搬迁安置，新时期精准扶贫扎实推进。大力发展文化事业，加快构建现代公共文化服务体系，实现基层公共文化设施建设全覆盖。保护传承西秦戏、正字戏、白字戏、汕尾渔歌等9个国家非物质文化遗产。连续举行五届市文化活动周和新年音乐会，成功举办两届广东省（汕尾）“马思聪杯”小提琴邀请赛、中国龙舟公开赛（汕尾海丰站）、红海湾全国帆船赛等全国性文体赛事，获得80多个省级文艺奖项，荣获“广东省小提琴艺术之乡”称号。深入创建平安汕尾，开展“雷霆扫毒”“飓风2016+”系列行动，陆丰制贩毒得到明显遏制。加强基层治理，妥善处置东涌、乌坎涉稳问题，实现“一村（社区）一法律顾问”，信访量逐年下降，社会治安稳定持续向好。积极推进地方立法，《汕尾市水环境保护条例》《汕尾市城市市容和环境卫生管理条例》两部地方性法规获准实施，《汕尾市城乡规划条例》立法工作有序推进。成功应对“天兔”“妮妲”“海马”超强台风等自然灾害，安全生产事故指标持续下降，连续五年没有发生重大安全生产和食品药品安全事故。我市实现“全省双拥模范城”三连冠，海丰县获“全国双拥模范县”称号。

此外，审计、统计、国防、人防、打私、气象、防震、台务、港澳、外事侨务、老龄、残联、民族宗教、档案修志、工会、青年、妇女儿童等工作取得新进步。

——过去五年，是政府自身建设不断加强、政务环境进一步优化的五年。我们始终把建设人民满意政府作为努力方向，对标广州、深圳等珠三角地区，不断加强政府自身建设。接受人大、政协监督，建立向人大报告工作和向政协通报工作的常态化机制，积极回应人大代表、政协委员对经济社会各方面问题的关注，五年办理市人大代表议案和建议125件、市政协提案506件。坚持从源头推动依法行政，严格规范性文件管理，修改、废止或宣布失效规范性文件139件。建立完善政府工作规则，正确履行政府职能。健全重大事项决策机制，提高决策透明度和公众参与度。深入推进政务公开，全面落实行政执法责任制，推行政府法律顾问制度，行政复议应诉工作不断加强。全面完成公车改革。持续加强作风建设，认真落实中央八项规定精神，扎实开展党的群众路线教育实践活动、“三严三实”专题教育及“两学一做”学习教育。深入开展政务整治、正风肃纪集中行动，从严查处“为官不为”问题和案件248件，处理538人，政务环境不断优化。

——过去五年，是深圳全面对口帮扶力度持续加大、深汕合作进一步深化的五年。深圳市认真贯彻落实省委、省政府对口帮扶的部署，提出打造深莞惠+汕尾河源“3+2”经济圈，累计投入帮扶资金56.4亿元。与汕尾共建“1+4”产业园区，组织两市2000多家企业参加“深汕百企牵手合作”产业对接活动，创办汕尾海洋文化旅游节，帮扶引进产业项目289个，为我市加快发展注入强劲动力。全力推动扶贫“双到”和精准扶贫工作，实施帮扶项目4300多个，被帮扶村人均收入和集体收入分别增长3倍和13倍，在省第二轮扶贫“双到”考核中排名第一。深圳市4个区、34个部委办局、10家国企倾情倾力对口帮扶汕尾，413名深圳干部驻扎汕尾长期帮扶，不仅为汕尾带来实实在在的经济社会发展，还带来先进的理念和优良的作风。

我们深切地感受到，汕尾的每一个变化、每一个进步，离不开省委、省政府和市委的正确领导，凝聚着全市360万干部群众的勤劳和智慧，倾注着社会各界的关心和支持。在此，我代表市人民政府，向广大工人、农民、知识分子、干部职工，向驻汕人民解放军、武警官兵和各民主党派、人民团体、外出乡贤、社会各界人士致以崇高的敬意！向长期关心支持我市经济社会发展的港澳同胞、台湾同胞、海外侨胞和国际友人表示衷心的感谢！

我们清醒地认识到，发展中还存在不少矛盾和困难：一是振兴发展基础较差，经济欠发达、发展不足仍是最大的市情，人均生产总值长期低于全国、全省平均水平，实现全面小康任务重、压力大。二是实体经济规模小，产业集聚水平低，规模以上企业少，创新能力不足，发展动能亟待提升。三是城市发展水平不高，中心城区首位度低，城乡差距较大，交通秩序、市容市貌、生态环境有待进一步改善。四是社会事业短板多，社会治理水平不高，基本公共服务均等化有待加强，人民群众高度关注的教育、医疗、社会保障等方面还存在较多问题。五是维护社会稳定压力加大，陆丰禁毒拐点虽已显现，但离摘除“毒帽”要求仍有差距，农村基层治理能力薄弱，乌坎村去年再次发生涉稳问题，产生了较大的负面影响。六是政务环境仍需进一步整治，政府办事流程不优，办事效率不高，一些干部干事创业的担当意识、责任意识不强，不想为、不敢为、不善为甚至乱作为的问题还不同程度存在。我们要以问题为导向，采取更加有力的措施加以解决。

未来五年总体思路

市第七次党代会提出了“建成宜居宜业宜游现代化滨海城市”的奋斗目标，对汕尾各项事业发展作出了全面部署，为政府工作指明了方向。

未来五年，市政府工作的总体思路是：高举中国特色社会主义伟大旗帜，全面贯彻落实党的十八大和十八届三中、四中、五中、六中全会精神，深入学习贯彻习近平总书记系列重要讲话精神，统筹推进“五位一体”总体布局，协调推进“四个全面”战略布局，坚持稳中求进工作总基调，牢固树立和贯彻落实新发展理念，适应把握引领经济发展新常态，以提高发展质量和效益为中心,以供给侧结构性改革为主线，坚定向西融珠发展，推进深汕产业共建，全力扭住“三大抓手”、做强“三大产业”、打造“四大环境”、攻坚“四大民生”，把汕尾建设成为宜居宜业宜游现代化滨海城市，打造区域协调发展广东样本，实现全面建成小康社会的目标。

未来五年，我市经济社会发展主要目标为“一个确保、四个显著”。“一个确保”就是确保如期全面建成小康社会，经济保持高于全国、全省平均水平的中高速增长，发展迈入中高端，2020年与全国同步全面建成小康社会，到2021年，地区生产总值突破1500亿元，年均增长11%，人均生产总值达48000元。“四个显著”就是产业集聚显著增强、民生福祉显著改善、城市形象显著提升、基层基础显著夯实。实现上述目标，必须把思想和行动统一到市第七次党代会的决策部署上来，准确把握汕尾发展所处的历史方位和阶段特征，在新的起点上谋求新发展、实现新跨越、创造新辉煌。

一是要把握三大机遇，增强发展信心。未来五年，经济基础薄弱，基础设施欠账多，社会民生落后仍然是影响和制约汕尾发展的突出问题。但同时，我们也迎来了“三大发展机遇”。省委、省政府着眼区域协同发展大局，大力实施促进粤东西北振兴发展战略，谋划构建大珠三角经济区，突出产业共建，推动珠三角9市与环珠6市一体化发展，确定深圳全面对口帮扶汕尾，深圳认真贯彻落实省委、省政府决策部署，积极实施东进战略，主导推动深莞惠+汕尾河源“3+2”经济圈建设，为我市产业、交通、城市发展带来新的契机。随着供给侧结构性改革的不断深入，加快补齐基础设施和民生短板，大幅降低制度性交易成本和各种要素成本，为我市调结构、促转型、稳增长、提质量找到了重大突破口。海陆丰革命老区纳入国家贫困革命老区扶持范围，国家、省在财政、投资、金融、土地、生态补偿等方面给予我们一系列特殊扶持政策，同时，众多外出乡贤热切关心家乡建设，越来越多客商到汕尾投资兴业，为我市新一轮发展注入强大动力。只要我们牢牢把握并抓紧用好这些重大机遇，顺势而为、乘势而上，就一定能够克服重重困难，实现快速崛起。

二是要强化四个意识，不忘改革初心。为实现市第七次党代会描绘的宏伟蓝图，政府要以“四个强化”改变“思想意识、能力水平、纪律作风、工作落实”四个滞后。必须强化争先意识。传承和发扬汕尾人敢为天下先的精神，激发打翻身仗的士气，不待扬鞭自奋蹄，坚决摒弃甘居人后的惯性思维，破除“等靠要、推拖慢”等不良习气，牢固树立“今日事今日毕，明日事今日计”的紧迫意识，推动各项工作争先进位，确保经济社会发展主要指标、各项考核排名前移，在粤东西北12市中加快突围，实现全面小康目标。必须强化质量意识。牢固树立崇尚质量、追求卓越的思维与理念，深入实施质量强市战略，坚持速质兼优，追求高质量的经济发展，使产业更优、内生动力更足；坚持以人民为中心，追求高层次的精神文明，使社会更加和谐，人民更加幸福；坚持以百年不落后为标准，追求高品位的城市发展，使城市布局更加合理，功能更加完善，发展更富内涵；坚持绿色发展，以对子孙后代负责的态度，坚决避免饥不择食，急功近利，宁愿发展慢一点，也要好一点，行稳致远，使生态更加美好，汕尾更加美丽。必须强化实干意识。进一步解放思想，大干快上，敢于打破“旧框框、老套路”的重重束缚，破除“差不多、过得去”的粗放思维，转变“光说不练、做而不实”的漂浮作风，树立一抓到底的实干精神，以抓铁有痕、踏石留印的劲头，撸起袖子加油干，以优良作风把汕尾的工作做实、把汕尾的事情办好。必须强化担当意识。始终保持奋发有为的精神状态，正视问题不回避，承担责任不推诿，以逢山开路、遇水搭桥的智慧和勇气，知难而进、迎难而上，敢于碰硬、勇于担当，集中力量突破重大瓶颈制约，带领群众闯出发展新路子。

三是要打赢六场硬仗，坚定发展决心。落实市第七次党代会提出的“三大抓手、三大产业、四大环境、四大民生”，关键是要全力打赢六场硬仗。打赢产业共建的硬仗。汕尾振兴发展最重要最核心的要靠两条：一要靠引进工业项目，成为珠三角和深圳的制造业生产基地；二要靠发展旅游休闲、健康养生等生产性服务业，成为珠三角和深圳的后花园。这些都需要产业共建来实现。省委、省政府为深化珠三角地区和粤东西北全面对口帮扶，提出把产业共建摆在更加突出位置，推动更多珠三角优势产业、优质企业把生产环节布局到粤东西北，形成紧密协调的产业分工体系。我们要紧紧抓住这次机遇，坚定向西融珠发展，主动对接珠三角优化发展战略、深圳东进战略，主动参与深莞惠+汕尾河源“3+2”经济圈建设，主动推进与珠三角交通、产业、政策和基本公共服务一体化发展，实现要素资源自由流动、优化配置。要以深汕特别合作区为引领，按照“总部、研发、销售在深圳，生产基地在汕尾”的产业共建新模式，大力推进产业园区扩园建设、提质增效，力争到2021年“1+4”产业园区规划面积达151平方公里，建成面积78平方公里以上。加快园区产业集聚，每年推动不少于30个投资超亿元的工业项目落地建设，力争到2021年承接珠三角和深圳转移产业项目300个以上，规模以上工业增加值达600亿元以上。打赢提升中心城区首位度的硬仗。明确市中心城区发展定位，优化空间布局，有序扩大规模容量，增强人口吸纳、要素集聚和辐射带动能力，力争到2021年，中心城区建成面积达70平方公里，常住人口达72万人以上，人均生产总值达10万元以上。加快市中心城区“东扩、西延、北拓”，东部加快中央商务区建设，并向红海湾经济开发区和赤坑、可塘镇扩展，西部推进金町湾滨海旅游度假区和马宫特大中心渔港建设，并向深汕特别合作区延伸，北部以红草高新技术园区为节点，推进市区与海城双向拓展聚合，进一步拉开城市框架。优化提升市中心城区路网，构建以市中心城区为枢纽的交通体系。加快中心城区产业集聚，做大做强先进制造业、现代服务业，强化中心城区公共服务功能，大幅提升教育、医疗、文化、体育等发展水平和对全市的引领作用。同时，加快深汕特别合作区和各中心县城城市化发展步伐，使之成为辐射带动区域和县域经济发展的龙头。深汕特别合作区要充分发挥东进西融的尖兵作用，集中力量高起点高标准开发建设鹅埠、小漠片区，狠抓产业项目落地投产，承接深圳部分港口、物流基地、专业市场和职业教育、医疗养老等城市公共服务功能疏解，推动合作区与深圳同城化、一体化发展，努力打造“产业高地、生命绿洲、未来之城”。海丰县城要加快推进“三道两环两城两园一中心”建设，加速县城与市中心城区同城化进程，打造宜居宜业宜游现代化中等城市。陆丰市区要围绕“一中心两主轴六片区”，加快西移东扩、沿江发展，建设绿色、智慧、人文的现代化滨海中等城市。陆河县城要围绕“一轴三圈”战略布局，致力推进精致、精品县城和美丽乡村建设，全力打造优雅陆河，实现绿色崛起，建设客家新山城。红海湾经济开发区要围绕“一区一园一湖一港一路”建没，突出遮浪、田寮湖、田墘新城片区综合开发，创新“旅游+”业态，打造国际旅游休闲度假基地。华侨管理区要围绕“一城两园”，建设汕尾东部区域中心镇区，打造“绿色视界、善美侨区”。在做大做强中心城区和中心县城的同时，要做好中心镇总体规划修编工作，推进镇区综合整治，加强基础设施和公共服务能力建设，积极引导村镇二、三产业向中心镇集聚，促进当地农业转移人口就近就地城镇化。打赢创新驱动的硬仗。把创新作为引领发展的第一动力，主动融入珠三角区域协同创新体系，推动珠三角科技成果在汕尾产业化。实施高新技术企业培育、新型研发机构建设、企业技术改造、孵化育成体系建设、创新人才队伍建设、科技金融融合等“六大抓手”，推动创新驱动落到发展上，到2021年，R&D占GDP比重达1.6%，国家高新技术企业达120家以上，高技术制造业增加值占规模以上工业增加值比重达50%以上，工业技改投资年均增长13%以上，专利申请量、授权量分别达3500件、2800件以上。推进创新载体和平台建设，加快建设国家级和省级创新平台，新增各类研发机构、省级工程中心、实验室等120家，建设科技企业加速器10个、孵化器15个、创客空间20家，推动“双创”“四众”联动发展。坚持人才优先发展，大力实施“红海扬帆人才计划”，对引进的高层次人才实施补贴政策，规划建设“研发机构聚居园”和一批“人才公寓”，加强“人才驿站”和“博士、博士后、院士工作站”建设，打造一支规模较大的创新型人才队伍。打赢扩大有效投资的硬仗。振兴发展最终都要落实到项目投资上，今天的投资是明天的产出和民生福祉，没有投资就没有发展，就没有出路。我们要始终坚定不移抓好投资，未来五年，累计完成固定资产投资6000亿元以上，实现翻番目标。要优化投资结构，扩大有效投资，使工业、基础设施、房地产等投资保持在合理区间，降低投资风险，促进经济良性发展，避免出现投资泡沫和低效投资。要坚决打破“有多少钱办多少事”的思维定势，树立“按需求定项目再找钱”的新理念，各地各部门要按照经济社会发展、城市功能完善、民生福祉等现实需要来筛选投资项目，每年实际安排的项目要尽量往“五类项目”上靠，即产业等能挣钱的项目，交通民生等刚需项目，有现金流可以吸纳社会资本的项目，上级有扶持的项目，国家、省重点支持的项目。要深化投融资改革，解决项目资金从哪里来的问题，重点是打通“五大投融资渠道”，即国家政策性银行成本低期限长的扶持贷款，PPP等社会资本，政府债券、专项建设基金，与银行及非银行金融机构的合作，企业债。同时，创新投融资运作模式，建立“一办一库一平台一通道”，即成立投融资领导小组及其办公室，建立投融资项目库，组建水务、交通、城建等投融资平台，优化投融资审批通道。要想方设法增加土地收储，政府融资要优先用于土地收储，手中有适度规模收储土地才能为建设项目的投融资提供更大的腾挪空间。要围绕“精品、进度、成本”六个字狠抓投资效益，精打细算、按时保质做好每一个项目，尽可能不留遗憾。要做好项目前期工作，很多项目不能如期动工建设都是前期工作不充分、临时抱佛脚造成的，今后每年都要提出前期项目清单，安排专项资金，组织专业、精干力量认真抓好，彻底改变前期工作滞后的局面。要突出抓好现代化基础设施投资，继续全力开展交通建设大会战，构建陆海空交通体系，五年计划投资交通基础设施项目800亿元，重点加快广汕高铁、深汕高速公路拓宽工程、珠东快速、沿海景观快速路等交通重点项目建设，统筹推进国、省道等主干公路改扩建，力争全市公路通车里程突破6000公里，其中高、快速路300公里以上。加快小漠港区、湖东港区等深水码头建设，谋划开启汕尾海上客运航线，积极推动汕尾机场建设。统筹推进水电气讯等城乡基础设施建设，实施一批中小河流治理项目，全面完成村村通自来水工程，提高老城区饮用水质量。改造升级城乡供电网络，加快燃气管网建设，实施市生活垃圾无害化处理中心二期扩建工程，建设生活污水处理厂39座、农村污水处理设施1516个，实现城乡生活污水、垃圾收集基本全覆盖。加快4G基站等信息基础设施建设，无线宽带网络覆盖率达98%以上，光纤入户率达80%以上，城镇公共区域实现WLAN全覆盖，打造智慧城市。突出抓好教育、医疗、文体、住房等公共设施投资，不断提升公共服务水平。突出抓好产业投资，加快工业项目落地，力争制造业投资占固定资产投资比重达20%。打赢民生攻坚的硬仗。深入实施教育、医疗卫生、扶贫脱贫和农村基础设施“四大民生”攻坚，着力补齐民生短板，基本公共服务支出占一般公共预算支出比重达35%以上。打好教育攻坚战，以教育“创强争先建高地”为总抓手，到2018年和2020年，全市推进教育现代化先进县（市、区）覆盖率分别达50%以上和100%，教育优质化、信息化、多样化、国际化水平大幅提升。针对公益性和普惠性学前教育资源不足、义务教育城乡差距较大、大班额现象较为突出、高中阶段教育普职不协调及特色不明显、特殊教育资源不足、高等教育薄弱、教师队伍结构性缺编和教育质量整体不高等突出问题，以推进幼儿园规范化建设、义务教育学校现代化建设、普通高中多样化有特色发展、中等职业学校内涵优质发展、特殊教育提升发展、高等教育加快发展为基础，以完善基层教育治理机制和建设高水平校长、教师队伍为重点，加强教育教学管理，切实提高教育质量和办学水平。打好医疗卫生攻坚战，深入实施医疗卫生强基行动计划，稳步推进医疗资源结构优化和布局调整，全面深化公立医院改革，加快乡镇卫生院标准化和村卫生站规范化建设，力争创建5家三甲医院，建成汕尾市中心医院，每千人床位数、执业（助理）医师数、注册护士数分别达4.2张、2.8人、3.5人。打好扶贫攻坚战，全面对接深圳对口帮扶，大力实施精准扶贫，2018年全市13.4万相对贫困人口全部脱贫。加快农村基础设施建设，逐步实现城乡居民生活条件同质化、公共服务均等化，让农村成为农民幸福生活的美好家园。打赢重塑形象的硬仗。春华书记一针见血地指出，形象不佳是影响粤东地区发展落后的重要原因。我们要知耻后勇，树立新风正气，重塑汕尾正面形象。全力推进“创文”工作，确保跻身广东省文明城市行列。大力弘扬“包容守信务实争先”的城市精神，扎实开展“汕尾处处有雷锋”主题活动，形成良好的社会风气。大力整治市容市貌，打造干净整洁有序的城乡环境。以建设平安汕尾为目标，重拳整治涉毒、涉枪、涉盗抢、涉电信网络诈骗、涉黑恶等突出违法犯罪，全力实现陆丰涉毒重点整治地区摘帽目标，加强基层治理，打造平安和谐稳定的社会环境。全力推动政务整治，加强政府廉政建设，打造良好的政务环境。深化“放管服”改革，树立市场意识、诚信意识、法治意识和国际化理念，打造良好的营商环境。坚决守住生态底线，加强生态环境治理和保护，开展新一轮绿化大行动，使汕尾青山常在、清水长流、空气常新。这些硬仗涉及经济社会的各个方面，任务非常艰巨，责任重于泰山。只要我们下定决心，坚定信心，一步一个脚印、一年一个台阶，坚定不移地打好攻坚战、持久战，就一定能够战胜各种困难，让汕尾振兴发展谱写新的篇章。

2017年工作安排

今年是新一届政府的开局之年，是实施“十三五”规划的重要一年，也是供给侧结构性改革的深化之年，做好各项工作意义重大。今年经济社会发展的预期目标：地区生产总值增长8.0%，人均生产总值增长7.0%，规模以上工业增加值增长10.0%，固定资产投资增长18.0%，社会消费品零售总额增长9.8%，外贸进出口总额增长8.0%，一般公共预算收入增长10.0%，城乡居民人均可支配收入增长8.4%，居民消费价格涨幅控制在3.0%以内，城镇登记失业率控制在3.0%以内，节能减排约束性指标完成省下达年度任务。

实现上述目标，重点抓好八个方面工作：

一、全力扩大有效投资，增强经济发展动能

今年计划列入省重点项目36个，年度投资179.7亿元；安排市重点项目179个，年度投资320.2亿元，带动固定资产投资完成790亿元。

——加快基础设施建设。计划投资94.9亿元，全面推进交通、发展平台、农林水利、市政建设等基础设施建设。加快推进广汕铁路、兴汕高速动工建设，做好深汕高速改扩建、珠东快速（市区至陆丰、海丰）、沿海景观快速路、汕尾机场等前期工作，改扩建海汕公路红草至西闸段、国道324海丰县城至梅陇和县城至可塘及陆丰穿城路段、国道235陆河东互通至河口段、海丰莲花大道等一批县域道路。加快深汕特别合作区、汕尾新区、海丰、陆丰、陆河产业园区和红海湾滨海旅游区等基础设施建设。推进小漠国际物流港、马宫和遮浪现代渔港等项目建设，抓好54公里海堤达标加固、73.6公里中小河流治理等水利基础设施建设。推进城乡电网、宝丽华电厂出线走廊及一批输变电站等电力基础设施建设。加快高速宽带、泛在移动、天地一体、智能便捷等新型信息基础设施建设，新增光缆线路3020皮长公里，新建4G基站1220座，实现市区重点公共场所免费WLAN全覆盖，加快智慧城市进程。抓好中央预算内投资项目建设。

——加快产业项目投资。推进陆丰核电、甲湖湾电厂、陆河和红草比亚迪项目、信利TFT5代线、和合锂电、星际动漫、海王制药、香雪制药、百草堂中药饮片、路华电子、金科特种材料、易能电气、乾泰电池、康强管道、华侨古建筑材料市场等项目建设。推动重大工业项目加快投产达产，力争全年完成工业投资190亿元。推进新一轮企业技术改造，力争全年完成技改投资65亿元。大力扶持民营经济发展，建立市级财政专项资金，实施中小企业成长计划，推动“个转企”“小升规”，确保全年新增“四上企业”100家以上。

——强化投资保障。积极扩大投融资规模，大力拓宽投融资渠道，想方设法为项目寻求成本低期限长的资金。加强土地收储，今年全市完成征地3万亩，为投融资改革提供支撑。落实一项目一领导，一项目一时间、责任、问题清单，一月一督查等“三个一”工作机制，加强投资项目考核，确保项目尽快动工投产，发挥最大的效益。

二、全力推动产业共建，提升实体经济发展层级

——进一步促进园区提质增效。抢抓新一轮产业共建重大机遇，充分利用省普惠性和叠加性政策奖补措施，推动深圳扶持合作区产业政策覆盖全市共建园区，在土地使用、招商引资、技术创新等方面制定支持性政策，全面降低土地、税费、管理等制度性交易成本，大力承接珠三角产业转移。加快园区工业项目建设，确保入园动工工业项目62个，其中建成投产22个，实现工业产值600亿元以上，全口径税收27亿元。拓展园区发展空间，推动土规向产业园区倾斜，加快红草、陆河产业园向周边扩容，支持海丰、陆丰产业园发展“一园多片区”，全市园区征储土地2.4万亩。投入30亿元，完善园区基础设施及生活配套设施，加快公共检测服务平台建设。严格执行投入、产出、用地等刚性约束标准，促进园区绿色高效发展。聚焦鹅埠、聚焦产业，推动合作区大开发大建设，完成土地整备1.5万亩，加快合作区国道324市政化改造示范段及市政道路建设，创建国家自主创新示范区、全省产业共建示范区。

——全力推进招商引资。坚持大招商、招大商，实施“一把手招商”“一对一”精准招商、小分队行业招商和市场化中介招商。以珠三角地区大型骨干企业和特色优势企业的加工制造环节，大型骨干企业和特色优势企业增资扩产、转型升级项目，主导产业的配套企业，拥有较稳定国际市场份额的劳动密集型加工贸易企业，规模以上生产性服务业企业等为重点，精准开展产业对接，大规模、成建制、全链条引进更多的企业，推动项目加快落地投产，力争全年引进落地5000万元以上产业项目60个。

——加快发展现代产业。振兴实体经济，核心是加快制造业转型升级，实现新旧动能转换。必须大力发展先进制造业，发挥比亚迪新能源汽车、信利光电、海王制药等龙头企业带动效应，引进上下游关联企业，加快形成电子信息、新能源汽车、生物医药、新材料、装备制造等产业集群，确保制造业投资占全社会固定资产投资比重达15%以上。大力发展旅游、健康养老、IT服务、现代物流、金融等现代服务业，确保现代服务业增加值占服务业增加值比重达42%以上。着力打造“红、蓝、绿、古、特”精品旅游带，创建红宫红场—彭湃故居、水底山温泉旅游区两个4A景区，提升红海湾、凤山、铜鼎山、玄武山、莲花山“一湾四山”景区品质，启动海丰黄羌林场运动小镇、召贡山生态旅游和陆河白水寨瀑布等新景区规划建设。推进小漠物流港、红草南储冷链等项目建设，大力建设各类专业市场，加快发展现代商贸物流业。推进公平服装、可塘彩宝、梅陇金银首饰等产业园建设和陆河健康食品、城区海产品等传统优势产业发展，认真梳理其面临的困难问题，研究制订相应的扶持政策，推动转型升级，扭转下滑局面，加快纺织服装产业向时尚设计产业升级、金银珠宝首饰加工业向研发展销两端延伸，推动农副产品、海产品加工向“专精特新”发展。引导传统优势产业制订统一的标准，通过标准制订提升产业竞争力和品牌号召力。实施“互联网+”行动计划，推进互联网与各行业融合发展。

三、全力推进中心城区扩容提质，提高城市综合承载力

——以科学规划引领城市发展。立足大珠三角经济区全局，明确功能定位，对接深圳修编城市总体规划，推动汕尾与珠三角城市规划共绘，加快“多规合一”，确保“一张蓝图干到底”。做好交通、海岸线、城市更新等专项规划，完成市区西片、北片等控制性详规编制，抓好“可塘-赤坑-潭西”汕尾中心点、红海湾国际旅游休闲度假基地、品清湖区域空间发展和各中心县城等规划编制工作。

——统筹推进扩容提质。坚持扩容提质并重，提升市城区首位度和辐射带动力。加快市城区土地收储，完成征地1.6万亩。东片以中央商务区为中心，加快站前路、站前横路、东城大道东段等建设，拓宽红海东路，实施海绵城市样板工程，推进碧桂园时代城、保利大都会和汕尾农商行总部大厦等商贸综合体建设，推动物流、商贸、金融、会展等现代服务业集聚发展，打造城市新区。西片以一港一湾为重点，加快工业大道西、金鹏路、西城大道、新衢路建设，扩建汕马路，启动建设罗马广场至沙滩公园海滨大道西段，推进马宫特大中心渔港和金町湾滨海旅游度假区建设，打造“丝路渔都”和城市旅游综合体。北片以红草高新园区为载体，推进红草大道、红草中路等园区路网和生活配套设施建设，加快产城融合，打造高端产业新城。中部加快新中轴线规划建设和环品清湖综合整治开发，推进环品清湖路东南段建设，实施环湖路绿化景观升级改造，形成“移步换景”的精品空间。推进品清湖蓝色海湾整治和美丽海湾建设，完成环品清湖7个重点排污口截污工程和避风塘湖底清淤疏浚、防波堤改造，全面改善品清湖水质和沿岸环境。推进老城区城市更新，争取和顺上村和万达广场城市更新项目上半年动工建设，加快其他城市更新项目前期工作，结合城市更新逐步推进四马路升级改造和打通二马路、兴华路等工程。启动南区污水处理厂一期建设工程，以承接东区污水处理厂搬迁。加快市区公交场站、公园、停车场、公厕、文体设施、垃圾转运站、人行天桥、余泥渣土受纳场建设，完善城市功能。

——全面加强城市管理。以“创文”“创卫”为目标，全面开展城乡环境整治行动，向“脏乱差堵”宣战。持续严厉打击违法建设，确保市区违建“零增量”。深入推进干线公路、背街小巷、集贸市场卫生环境综合整治，清理整顿占道经营、乱停乱放，全面清理违章户外广告，落实“门前三包”责任制。坚决依法取缔非法营运三轮车，妥善解决好三轮车从业人员再就业和特困人员生活保障，增加新能源纯电动公交车至600辆，实现运营时间和运营线路两个全覆盖，满足市民出行需求。完善城市管理和综合执法体制，推动执法重心下移和属地化管理。创建文明村居523个、文明镇（街道）35个，市中心城区进入省“创文”先进城市行列，其他县（市）进入省“创文”先进县城(城市)行列。

四、全力推进改革创新，加快对外开放合作步伐

——系统推进全面创新。今年市财政安排2亿元专项资金用于实施创新驱动战略，推动以科技创新为核心的全面创新。力争国家高新技术企业达30家，R&D占GDP比重提高到1%。推动区域协同创新，面向珠三角跨区域整合创新资源布局。规划建设科技企业加速器、孵化器、“双创”基地，全市孵化面积达9.5万平方米。加强与珠三角科技金融机构合作，设立风投基金和产业引导基金。加快创新科技载体建设，推进深汕特别合作区申报国家自主创新示范区、汕尾高新区申报国家级高新区，新增各类研发机构、研究中心、实验室等36家，新建创客空间6家，吸引创新创业团队和1000名以上创客进驻。鼓励自主创新，争取专利申请量达1400件、授权量达960件以上。实施“红海扬帆人才计划”，设立2亿元的“红海扬帆人才基金”，今年市财政首期注资5000万元，用于引进高层次领军人才、创新创业团队、硕博毕业生、名师名医等的奖励和补贴，今后硕士和博士研究生毕业到汕尾工作，分别一次性补贴2万元、5万元。

——全面深化改革开放。深入推进供给侧结构性改革，落实“三去一降一补”任务，基本实现“僵尸企业”市场出清。深化行政审批制度改革，全面完成行政许可标准化改革，继续推广“一门式、一网式”政府服务模式，推动负面清单全覆盖。深入推进商事制度改革，落实“双随机一公开”制度，推进企业“多证合一”。做好营改增改革的各项工作。按照中央和省的部署，推进国资国企、产权保护、价格财税金融、对外开放、养老保险等重要改革。积极参与国家“一带一路”建设。主动融入大珠三角经济区，全面落实深莞惠+汕尾河源“3+2”党政主要领导第十次联席会议涉及我市的重点合作事项及《海洋产业经济协作示范区建设纲要》《共建海上旅游航线发展粤东滨海旅游框架协议》。加快外贸转型升级示范基地和公共服务平台建设，推进口岸“单一窗口”建设和通关一体化，促进外贸发展提质增效。

五、全力推进农业供给侧结构性改革，引领“三农”工作转型升级

——加快发展现代农业。以农业供给侧结构性改革为主线，把增加绿色优质农产品供给放在突出位置，推动质量兴农。持续夯实现代农业基础，新建高标准农田11.94万亩、粮食仓容10万吨，加快海丰优质水稻生产基地建设，支持华侨区建设省良种良法示范基地，保障粮食安全。扶持国泰、海亮、陆港等农业龙头企业做大做强，争创国家级农业标准化示范区1个、省级农业标准化示范区4个，提高农业产业化水平。推进农产品冷链物流体系建设。依托“互联网+农业”新业态，大力发展农村电商，完善农村现代物流服务体系。大力发展观光农庄、村镇旅游、农家乐等休闲农业,抓好海丰县平东农业主题公园和莲花山休闲农业观光园、陆河县罗洞生态农业观光园、华侨管理区四季果园旅游区等建设。加快马宫特大中心渔港项目建设，培育远洋捕捞船队，加强海洋生物医药研发，发展海产品精深加工，提升海洋渔业发展水平。

——深化农业农村改革。深入推进农村集体产权制度改革，细化落实承包土地“三权分置”办法，全面铺开农村土地承包经营权确权登记颁证工作，确保年底前完成70%确权任务。完善农村集体“三资”管理服务平台建设，实现县、镇、村三级互联互通。推进供销社综合改革。加快农村金融改革创新，引进1家村镇银行，提升农村普惠金融“村村通”水平。

——加快推进新农村建设。开展村庄规划编制工作，规范和引导提升村庄建设。完成农村公路建设59公里，确保2018年底基本实现村村通客车。继续推进村村通自来水工程建设，推动村村通光纤、村村覆盖4Ｇ网络。加快农村电网改造升级。加强农村人居环境集中连片整治，创建一批宜居示范村庄。加快农村危旧房、“空心房”改造工作。发挥省级新农村连片开发示范效应，带动全市新农村建设。

六、全力补短板惠民生，增进人民群众获得感

——实施新一轮精准扶贫。把精准脱贫作为保障民生的首要任务，全面落实“六个精准”要求，实施“五个一批”工程，确保今年5.7万相对贫困人口实现脱贫。突出产业扶贫，引导贫困村发展农产品加工业等特色产业，以参股分红的方式发展物业。加大贫困人口劳动技能培训力度，建立与比亚迪、信利等大型企业定向劳务输送制度，促进贫困人口就业。落实教育扶贫资助、基本医疗保障、农村低保兜底脱贫等政策，提高贫困人口社会保障水平。主动对接国家和省革命老区扶贫政策，争取上级在财税、投资、金融、国土、生态等方面更多扶持政策落地。全面对接深圳对口帮扶，统筹推进以产业共建为重点的全面对口帮扶和以脱贫攻坚为重点的扶贫“双到”工作。

——深入推进教育攻坚。全面启动教育现代化先进市创建工作，确定时间表、路线图，强化督导考核，推动陆河县率先建成教育现代化先进县。大力建设以公办园和普惠性民办园为主体的学前教育服务体系，新增幼儿园31所。实施新一轮中小学布局调整，新改建一批义务教育阶段学校，抓好义务教育“防流控辍”工作，确保初中辍学率控制在2%以下。推进市林伟华中学、市城区新城中学、陆河县河田中学创建国家级示范性普通高中，抓好陆丰市龙山中学新校区等项目建设，确保高中毛入学率达88.7%。推进汕尾职业技术学院扩建、市体校新校区建设工程，提升职业学校、技工学校办学水平。深入实施“强师工程”，新增5个以上市级教师工作室，健全校长竞争上岗制度，继续实施“百名研究生引进计划”和“千名教师本科学历提升计划”，试点推进市级教师发展中心和各县（市、区）教师进修学校建设。推进中心小学以上学校百兆宽带全覆盖。加大教科研工作力度，加强义务教育和高中教育教学质量监测。大力扶持民办教育、特殊教育发展。

——全力补齐医疗卫生短板。实施医疗卫生强基行动计划，全面推进分级诊疗制度建设，提升医疗卫生现代化水平。全力推进深圳援建的汕尾市中心医院项目建设，加快土地清理和建设设计，确保这个高水平的三甲医院今年上半年动工建设，提升市城区医疗卫生带动能力。深化与中信集团合作，力争市人民医院创建成为三甲医院。建设市第三人民医院和3所县级综合性医院，推进市第二人民医院肿瘤防治中心、海丰县彭湃纪念医院新院区和陆河县人民医院住院大楼等项目建设。对接省、深圳医院对口支援，大力引进医学优势学科和优秀医学人才，力争市人民医院、市第二人民医院、海丰县彭湃纪念医院、陆丰市人民医院各新增1个省级临床重点专科。全面深化县级公立医院综合改革，继续扩大基本药物制度实施范围，确保县域内住院率达90%以上。推进县级医院与乡镇卫生院一体化管理，完成23家标准化乡镇卫生院建设，提高村卫生站医生待遇。支持民营医院发展，形成多元化办医格局。加强疾病预防控制能力，完善公共卫生安全体系。建设市级卫生计生数据中心机房和卫生信息平台。开展新生儿出生缺陷综合防控、免费孕前优生健康检查，免费为全市7.6万农村妇女实施“两癌”检查。落实全面两孩政策，促进人口均衡发展。

——加快文体事业发展。精心办好“马思聪杯”小提琴邀请赛、F1摩托艇世锦赛、红海湾国际冲浪邀请赛、中国龙舟公开赛（汕尾海丰站）、市运会、市群众音乐舞蹈花会等文体赛事，打造文化惠民活动周、新年音乐会和“一县一品”群众文化活动等文化品牌。启动市图书馆扩建工程，加快推进市民文化中心建设，完成市民体育广场建设，完善基层文体设施配套。加强文物保护，注重非物质文化遗产的保护、传承与发展，创建文化旅游融合发展示范区。

——推进人社等社会事业发展。突出抓好高校毕业生、就业困难人员等重点群体就业，实现城镇新增就业4.8万人，新增转移农村劳动力就业2.7万人，城镇登记失业率控制在3.0%以内。推动城乡居民与城镇职工医保制度一体化，完善城乡居民基本养老保险制度。稳妥推进机关事业单位养老保险制度改革。优化大病保险制度，把困难群体报销比例提高到70%以上。加快“智慧人社”建设，创建国家社会保险标准化建设“先行城市”。加快养老服务体系建设，推进敬老院公建民营试点工作。继续深化殡葬改革，推进镇级公益性公墓（骨灰楼）建设，力争新增10个公益性公墓（骨灰楼）。

同时，注重解决民生突出问题，办好十件民生实事。

七、全力改善生态环境，推进绿色低碳发展

——大力加强生态环境治理和保护。全面落实大气污染防治行动计划，加强空气质量监测，确保空气质量持续向好。加强水污染防治，深入实施南粤水更清行动计划，推进奎山河、奎山湖治理，强化公平水库、赤沙水库、螺河等集中式饮用水源地保护区规范化管理，抓好公平水库灌区总干渠饮用水隔离保护工程。实施土壤污染防治和农村环境保护行动计划，促进土壤资源永续利用。推进市生活垃圾无害化处理中心二期工程、中心镇污水处理厂建设，加强垃圾填埋场治理，完善城乡垃圾收运系统，提升垃圾、污水综合处置能力。深入开展新一轮绿化大行动，加强汕尾大道、环品清湖等主干道和重点区域两侧山体绿化美化及林带林网建设，完成森林碳汇造林22万亩，建设生态景观林带50公里，新增森林公园3个、湿地公园 1 个，新增乡村绿化美化示范点100个。严守林业生态红线，严厉打击破坏森林资源违法犯罪行为，加快推进桉树退出。优化海洋生态环境，做好近岸海域污染防治、综合整治和生态修复，加强龟龄岛、遮浪岩等礁岛的保护。推进红海湾国家级海洋公园建设。

——强化节能减排降碳。强化约束性指标管理，确保完成省下达的节能减排降碳目标任务。加强高能耗行业管控，推进煤电超低排放。加强机动车污染防治，完成省淘汰“黄标车”任务。加快新能源汽车推广应用，投放运营新能源纯电动公交车600辆，推动全市0-50公里公交化全覆盖。实施全民节能行动计划，倡导绿色出行，推广建筑节能和绿色建筑。推进节约集约用地，加大闲置土地处置力度。

八、全力推进社会治理创新，维护平安和谐稳定大局

——加强城市公共安全建设。深化平安汕尾建设，完善立体化社会治安防控体系，持续开展社会治安集中整治，严厉打击涉毒涉枪、涉盗抢、电信网络诈骗等违法犯罪。以实现陆丰摘除“毒帽”为目标，推进禁毒重点整治行动，持续开展清查打击、堵源截流、宣传教育、综合治理，坚决打赢禁毒这场人民战争。市里把海丰列为今年全市社会治安重点整治地区，要加大工作力度，确保整治取得成效。建立覆盖城乡的“中心+网格化+信息化”工作体系，提高群防群治水平。吸取“乌坎涉稳问题”教训，深入开展社会矛盾纠纷排查，落实领导包案，化解信访积案，维护社会和谐稳定。实施“七五”普法，增强全民法治观念。加强城市安全和公共安全工作，提高突发事件预防预警和应急处置能力。落实安全生产党政同责、一岗双责、失职追责，加快安全生产监管信息平台建设，抓好重点领域事故隐患排查整治，坚决预防和遏制重特大事故发生。强化食品药品安全监管。加强财政风险防控，树立“过紧日子”的思想，该花的钱要大胆花，不该花的钱一分都不要花，厉行节约，压减一般性支出，确保财政平稳运行。加强金融监管，防止发生区域性系统性金融风险。做好国防动员、人民防空、双拥共建、优抚安置工作，巩固军政军民团结。

——加强基层治理。总结推广陆河基层治理试点县的经验，积极探索农村基层治理新路径。大力培育和规范发展自然村（村民小组）村民理事会，抓好村（居）务公开“五化”和村务监督委员会“九有”建设，推进社区协商、社区减负工作，推动公共服务进农村、进社区，全面提升基层自治能力和服务水平。完成村级换届选举工作，提高村“两委”干部素质和待遇。加快政社分开，培育和规范社会组织，推进政府向社会组织购买服务、转移职能。加快社工人才队伍建设，支持和发展志愿服务组织。

各位代表，推动今年经济社会加快发展，必须把全面从严治党的要求落实到从严治政中，坚持依法用权、廉洁从政、高效服务，加快建设为民务实清廉政府。推进依法行政。自觉接受市人大工作监督、法律监督和市政协民主监督。坚决执行市人大及其常委会决议决定，密切与市政协沟通协商，切实做好议案、建议和提案办理工作。认真听取各民主党派、工商联、无党派人士和人民团体的意见，主动接受群众、媒体和审计监督。深入推进政务公开和各领域办事公开。优化政务服务。健全各级政府权责清单制度，推动流程再造，探索推进并联审批等便捷服务。强化执行力建设。践行“三严三实”要求，全面对标广州、深圳等珠三角地区，树立“马上就办、办就办好”的服务意识，全面推行重大项目和重点工作限时办结制，完善全覆盖、常态化的绩效考核、审计监督和督查问责机制，以目标导向和倒逼机制推动工作落实。建立完善民生诉求快速回应和解决机制，做到“件件有落实、事事有回音”。深入推进反腐倡廉建设。坚决贯彻全面从严治党要求，坚持有腐必反、有贪必肃，坚持无禁区、全覆盖、零容忍，严肃查处各类违纪违法行为，重点查处不作为、慢作为、乱作为、“吃拿卡要拖”等问题，特别是“中梗阻”和窗口服务人员利用中介机构办事谋利问题。同时，建立有利于干事创业的激励机制和容错机制，努力打造一支政治坚定、敢于担当、勤政廉洁的干部队伍。

各位代表，全面建成小康社会，是新一届政府肩负的历史使命；人民对美好生活的向往，是我们履职尽责的最大动力。我们要更加紧密团结在以习近平同志为核心的党中央周围，在省委、省政府和市委的正确领导下，坚定信心、奋勇前行，为全面建成小康社会而努力奋斗，以优异成绩迎接党的十九大和省第十二次党代会胜利召开！
